# Beneficial fungi reprogram tree metabolism toward growth and stress-associated pathways

**DOI:** 10.1128/aem.00412-26

**Published:** 2026-06-18

**Authors:** Valiya Nadakkakath Agisha, Smitha Chandrasekharan, Savitha Dhandapani, Sng Yee Hwui, Lim Shi Hui, Somika Bhatnagar, Bong Soo Park

**Affiliations:** 1 Temasek Life Sciences Laboratory, National University of Singapore 37580, Singapore, Singapore; Michigan State University, East Lansing, Michigan, USA

**Keywords:** beneficial fungi, metabolomics, plant-fungus interaction, secondary metabolism, tree metabolism

## Abstract

Beneficial fungi are increasingly recognized as key modulators of plant growth and stress resilience, yet their functional roles in woody tree species remain poorly understood. Here, we examined the physiological, nutritional, and metabolic responses of sengon (*Falcataria moluccana*) to two plant growth-promoting fungi, *Penicillium olsonii* TLL1 and *Aspergillus aculeatus* TLL1, during a 6-month greenhouse experiment. Inoculation with *A. aculeatus* significantly enhanced leaf number, and shoot and root biomass compared with uninoculated controls, whereas *P. olsonii* showed more limited growth effects. Fungal inoculation led to a marked increase in root nutrient accumulation, including nitrogen (~2.5-fold), phosphorus (~1.5-fold), magnesium (~1.75-fold), and zinc (~2.5-fold), relative to controls, indicating improved nutrient acquisition. Untargeted metabolomic profiling revealed extensive metabolic reprogramming, with over 1,600 metabolites detected in negative ion mode and 2,800 metabolites in positive ion mode. Comparative analysis identified 367 and 206 differentially regulated metabolites in *P. olsonii-* and *A. aculeatus*-treated plants, respectively. Notably, enhanced nutrient status was accompanied by coordinated shifts in hormone-associated and secondary metabolic pathways, including phenylpropanoid-, lignin-, and terpenoid-related metabolites, consistent with a defense-primed metabolic state rather than constitutive defense activation. Together, these findings demonstrate that beneficial fungi reprogram tree metabolism by coupling improved nutrient acquisition with metabolic allocation toward growth and inducible defense, highlighting their potential role in sustainable forestry systems with tangible yield benefits.

**IMPORTANCE:**

Fast-growing tropical trees are central to reforestation and sustainable forestry, yet their productivity is often constrained by nutrient availability and vulnerability to pests and diseases. While beneficial microbes are widely used in annual crops, their functional impact on long-lived woody plants remains largely unexplored. This study shows that plant growth-promoting fungi do more than stimulate tree growth—they quantitatively enhance root nutrient acquisition and translate this nutritional advantage into systemic metabolic reprogramming. By integrating long-term growth measurements, elemental profiling, and metabolomics, we reveal that fungal colonization increases the bioavailability of key macro- and micronutrients in roots and supports the allocation of metabolic resources toward lignification, secondary metabolism, and inducible defense without compromising biomass accumulation. These findings provide mechanistic insight into how nutrient status and metabolism are coordinated in tree-microbe interactions and support the rational use of beneficial fungi in forestry and agroforestry applications.

## INTRODUCTION

Sengon (*Falcataria moluccana*, formerly *Paraserianthes falcataria*) is a fast-growing tropical tree species belonging to the Fabaceae (legume) family. Sengon is highly valued in forestry and agroforestry systems and is one of the preferred industrial forest plantations in Indonesia because of its rapid growth, adaptability to various soil types, and acceptable quality of wood (1, 2). Under good conditions, it can reach harvestable size within a few years, making it one of the most economically important short-rotation timber species. Sengon wood is generally lightweight and used for both the paper industry and furniture. As a nitrogen-fixing species, it is often used in reforestation programs, land rehabilitation, and agroforestry systems to improve soil fertility (3). However, sengon is susceptible to several pests and diseases, which can rapidly spread in tree plantations, severely reducing productivity (4, 5).

Plant growth-promoting fungi (PGPF) are known to influence plant development by enhancing nutrient availability, modulating phytohormone signaling, and reshaping host metabolic pathways through intimate plant-fungus associations (6). Species belonging to genera, such as *Penicillium*, *Aspergillus, Trichoderma*, and *Fusarium*, have been extensively studied for their growth-promoting and stress-mitigating effects in annual crops (7). In our previous study, we demonstrated the plant growth and phosphate solubilizing activity of *Penicillium olsonii* TLL1 (POT1) under phosphate-limiting conditions (8). The application of beneficial fungi has gained increasing attention as a sustainable alternative to chemical fertilizers and pesticides, with demonstrated benefits in crops, such as wheat (9), chili (10), tomato (11), and rice (12), etc. However, studies addressing the physiological and metabolic impacts of PGPF on tree species, particularly sengon, remain limited. Although endophytic fungi, including *Aspergillus* species, have recently been reported to promote sengon seedling growth, the underlying metabolic mechanisms governing these interactions are largely unexplored (13).

Despite growing recognition of the importance of beneficial microbes in plant systems, most mechanistic insights into plant-microbe interactions have been derived from herbaceous or annual model species. Long-lived woody plants differ fundamentally from annual crops in their carbon allocation strategies, developmental timescales, and reliance on secondary metabolism for structural reinforcement and long-term stress adaptation (14, 15). Consequently, microbial effects observed in herbaceous plants cannot be directly extrapolated to tree species, highlighting a critical knowledge gap in tree-microbe interaction biology.

Currently, a global overview of plant metabolomic mechanisms is accessible via high-throughput metabolomics studies. It is a powerful analytical approach for comprehensive investigation of cellular metabolites in various biological systems (16). In recent years, metabolomics has been increasingly applied to investigate plant-microbe interactions, providing system-level insights into how microbial colonization reshapes host metabolism (17). In tree species, metabolomic approaches have revealed substantial metabolic reprogramming during interactions with ectomycorrhizal and endophytic fungi, such as in oak colonized by *Pisolithus tinctorius* (18) and poplar colonized by *Laccaria bicolor* (19); such investigations have not been reported in sengon. Nevertheless, a few reports on phytochemical profiling have been available, and analysis of secondary metabolites revealed the occurrence of flavonoids, saponins, phenolic hydroquinones, tannins, triterpenoids, and steroids in susceptible and resistant sengon wood to gall rust disease (20). In another study, the anthelmintic activity of aqueous and ethanol extracts of *F. moluccana* bark waste containing tannins, flavonoids, alkaloids, saponins, and steroids was demonstrated (21).

A key unresolved question in plant biology is how plants coordinate growth with investment in secondary metabolism in response to microbial colonization. Secondary metabolites derived from phenylpropanoid, terpenoid, and shikimate pathways contribute to structural reinforcement, chemical signaling, and stress-associated responses, particularly in woody plants where lignification and long-term biomass formation are tightly linked to secondary metabolic flux (22, 23). Understanding how beneficial fungi influence this balance is essential for elucidating microbial strategies that enhance growth without compromising long-term metabolic investment.

Because fungal colonization can simultaneously affect nutrient acquisition, hormone-related metabolism, and secondary metabolic pathways, single-parameter physiological measurements are insufficient to capture host responses. Untargeted metabolomics provides an integrated framework to identify coordinated metabolic shifts across primary and secondary metabolism, enabling the detection of subtle but biologically meaningful reprogramming events that may underlie long-term growth outcomes in trees (17, 24).

In the present study, we investigated the effects of two dust-borne fungal isolates, *P. olsonii* TLL1 (POT1) (8) and *Aspergillus aculeatus* TLL1 (AAT1), on sengon growth under greenhouse conditions. AAT1 is a novel fungal isolate recovered from indoor house dust samples collected at Temasek Life Sciences Laboratory. Indoor dust represents an underexplored but ecologically diverse reservoir of filamentous fungi, encompassing both environmental generalists and taxa with demonstrated plant-associated functional traits (8). We reasoned that bioprospecting from this unconventional source would increase the probability of recovering functionally novel isolates with characteristics—such as stress tolerance and nutrient-mobilizing capacity—complementary to those of rhizosphere-derived strains. By integrating long-term growth measurements, elemental profiling, and untargeted metabolomics, we aimed to elucidate how fungal colonization reshapes tree metabolism and to identify metabolic signatures associated with enhanced growth and secondary metabolic investment. This work provides mechanistic insight into beneficial fungus-tree interactions and establishes a metabolomics-based framework for evaluating microbial inoculants in sustainable forestry systems.

## RESULTS

### Molecular characterization of fungus

AAT1 is distinct from the endophytic *Aspergillus* isolates recovered directly from plant tissue; here, AAT1 was characterized *de novo* using a five-locus molecular framework. The phylogenetic relationships between AAT1 and related fungal strains were inferred from maximum-likelihood trees using five molecular markers: internal transcribed spacer (*ITS*), large ribosomal subunit (*LSU*), translation elongation factor-1-α (*TEF1*α), beta-tubulin (*BenA*), and mini-chromosome maintenance protein (*MCM7*). The same or closely related *Aspergillus* species were clustered as a clade on the respective phylogenetic trees. The *ITS* sequence of AAT1 clustered with twelve other strains of *A. aculeatus* with a bootstrap value of 100 (Fig. S1). The use of other markers confirmed the accuracy in identifying the fungal species. The *LSU* and *TEF1α* sequences of AAT1 clustered with other isolates of *A. aculeatus* with bootstrap values of 99 and 88, respectively (Fig. S2 and S3). Additionally, the *LSU* and *BenA* and *MCM7* sequences of AAT1 were also grouped with other strains of *A. aculeatus* with bootstrap values of 78 and 65, respectively (Fig. S4 and S5). The species name of the fungus, AAT1, was assigned as *A. aculeatus* based on the clustering of related species in all the phylogenetic trees.

### Effect of fungal treatment on plant growth

**Fig 1:**
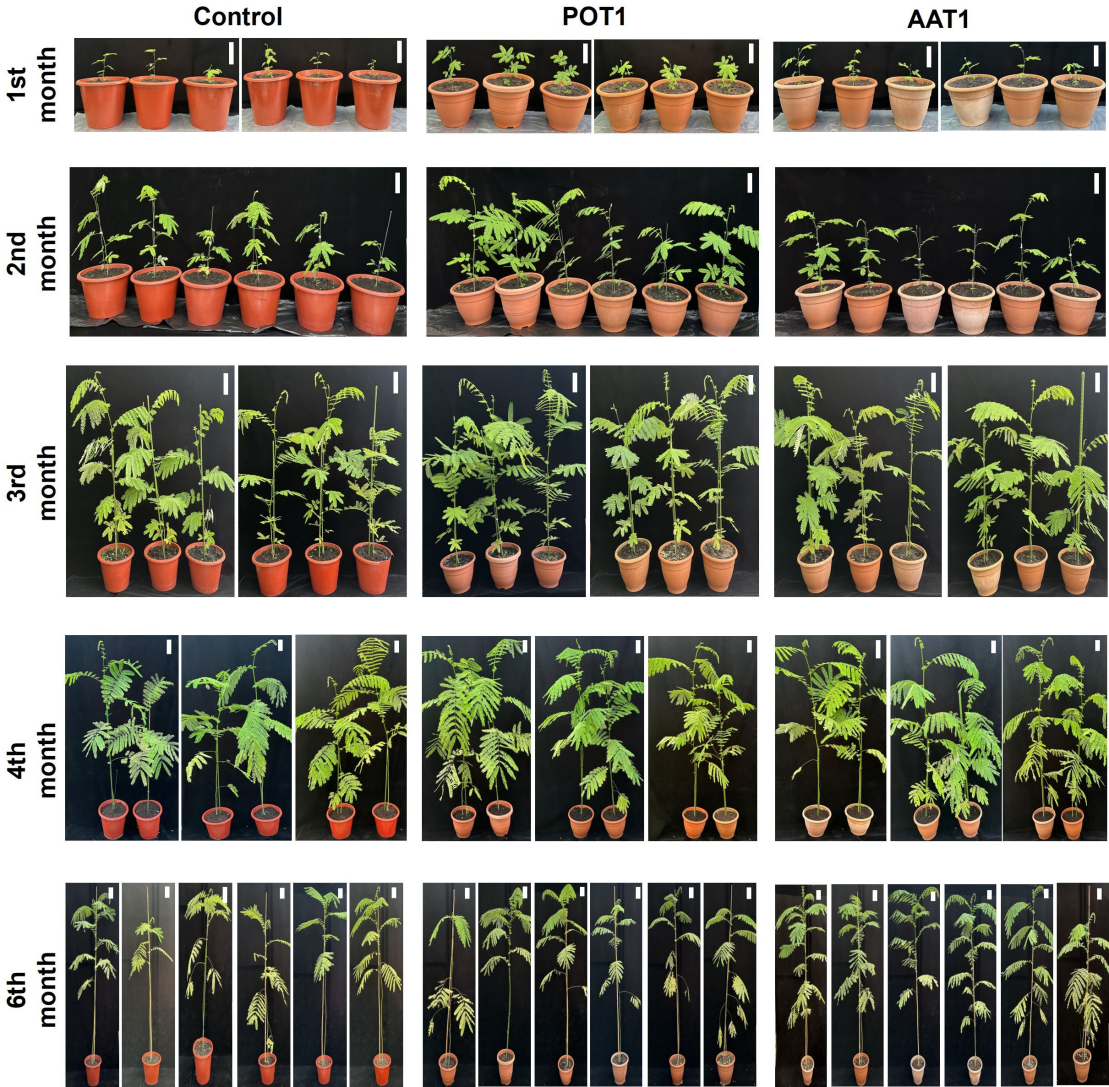
Effect of fungal colonization on sengon growth. One-month-old sengon seedlings were transferred to soil pre-inoculated with *P. olsonii* TLL1 (POT1) and *A. aculeatus* TLL1 (AAT1) and grown under greenhouse conditions for 6 months. One-month-old sengon seedlings were transplanted into the treated and control soil and grown under greenhouse conditions. Uninoculated soil served as control. Photographs illustrate control, POT1-, and AAT1-treated plants after first, second, third, fourth, and sixth month post-inoculation, displaying differences in vegetative growth. The scale bar represents 10 cm.

To assess the impact of fungal isolates on plant growth, sengon was cultivated in soil treated with POT1 and AAT1 (Fig. 1). The parameters, such as plant height and leaf number, were estimated at monthly intervals during the initial growth. POT1-treated plants showed increased plant height after 1st and 2nd months, while AAT1 had greater plant height only after first month compared with the control plants. Both POT1- and AAT1-treated plants did not demonstrate any significant difference in leaf number over a period of 4 months (Fig. S6 and Table S1).

**Fig 2:**
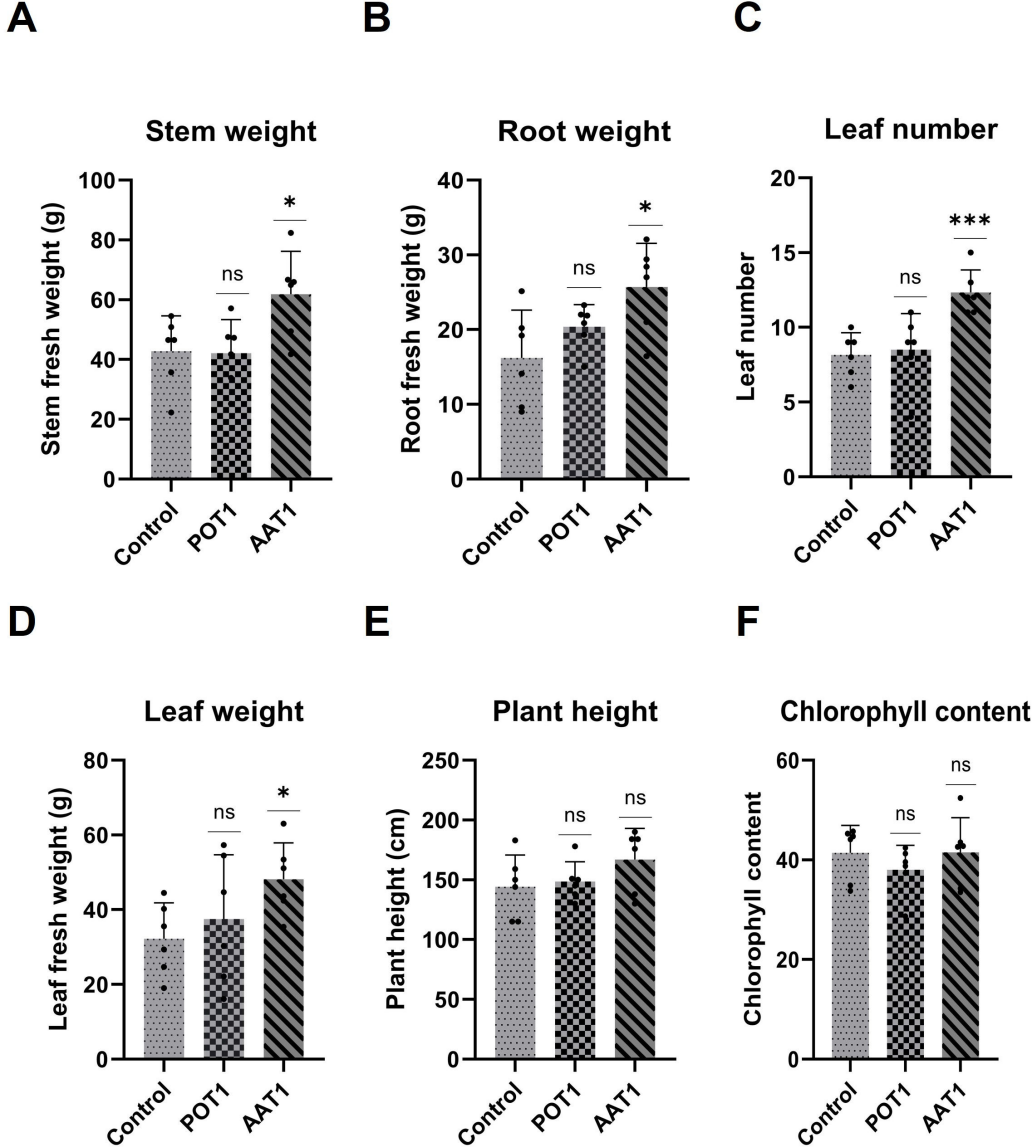
Effect of colonization on biological characteristics of sengon by *P. olsonii* TLL1 (POT1) and *A. aculeatus* TLL1 (AAT1). Quantitative analysis of various growth parameters: (**A**) stem weight, (**B**) root weight, (**C**) leaf number, (**D**) leaf weight, (**E**) plant height, and (**F**) chlorophyll content after 6 months post-inoculation. Data are presented as mean ± SD, with six biological replicates. Statistical significance was determined using an unpaired two-tailed *t*-test, with significance levels indicated by * *P* < 0.05, ** *P* < 0.01, *** *P* < 0.001; ns, non-significant.

After 6 months, AAT1-treated plants exhibited significantly higher shoot and root biomass, increased leaf number, and greater leaf weight compared with control plants. In contrast, none of these parameters showed a statistically significant improvement in POT1-treated plants relative to the control (Fig. 2A through D and Table S2). However, plant height and chlorophyll content did not vary between control and treated plants (Fig. 2E and F and Table S2).

### Colonization of sengon by fungal isolates

**Fig 3:**
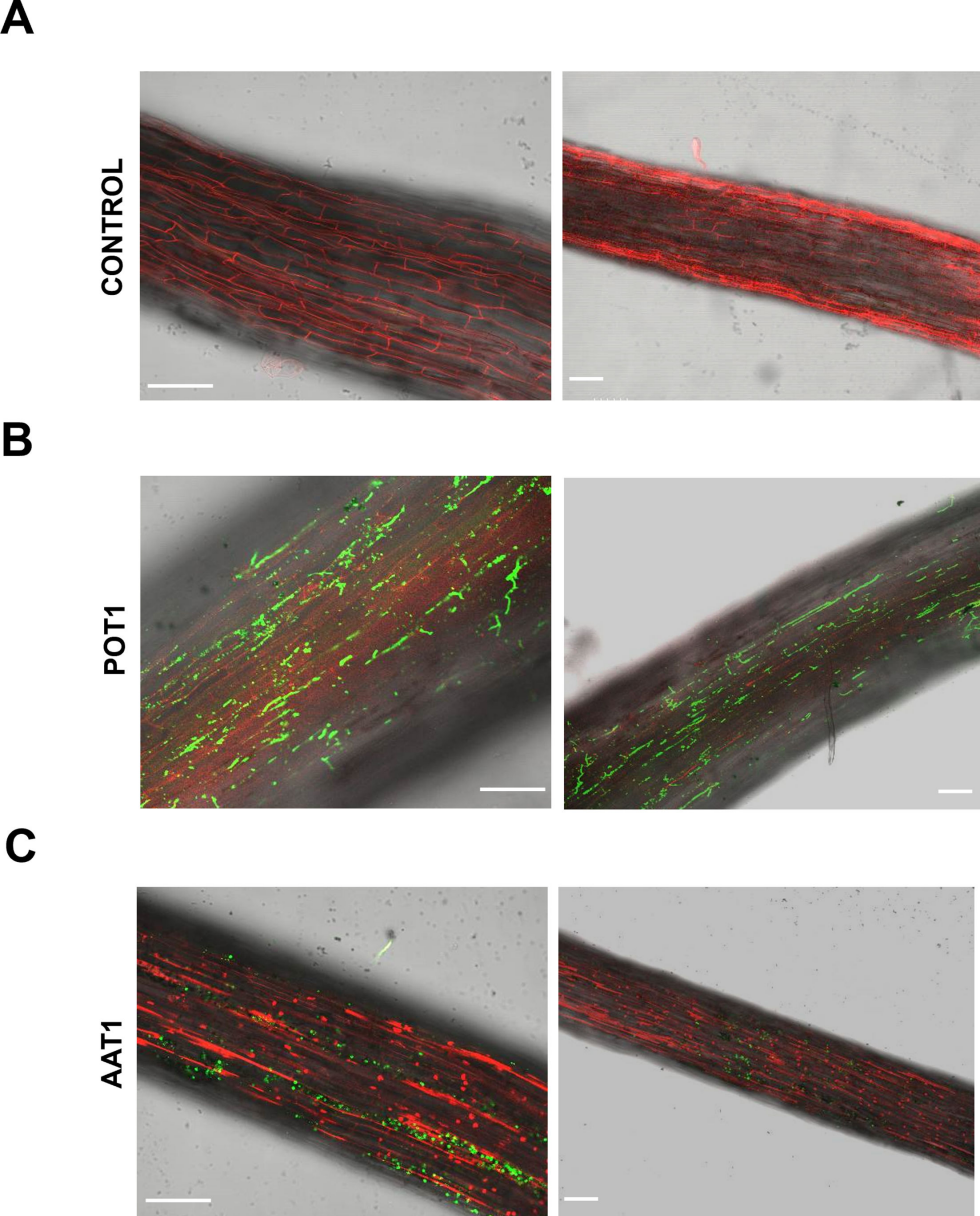
Colonization of sengon by *P. olsonii* TLL1 (POT1) and *A. aculeatus* TLL1 (AAT1). Representative images of WGA-AF488 and propidium iodide (PI) co-stained roots: (**A**) uninoculated, (**B**) POT1-treated, and (**C**) AAT1-treated. Fungal hyphae were stained with WGA-AF488 (green), and plant cell walls (red) were stained with PI. Scale bar represents 100 µm.

Fungal colonization in sengon was detected by staining using Agglutinin-Alexa Fluor 488 conjugate (WGA-AF488) and propidium iodide (PI). Microscopy analysis of the sengon seedlings co-cultured with the fungi, POT1 and AAT1, demonstrated that the fungi colonized the roots, and hyphal growth was observed on the root surface (Fig. 3). The two fungal isolates displayed distinct colonization architectures on sengon roots. POT1 formed extended longitudinal hyphae along the root epidermis, whereas AAT1 exhibited a more punctate, contact-point-rich pattern. Whether these architectural differences are further shaped by host root properties, such as cell wall composition or exudate profiles, remains to be determined, and future studies comparing colonization patterns across multiple host species will be valuable to disentangle fungal strain-specific from host-dependent determinants.

### Elemental analysis of sengon

**Fig 4:**
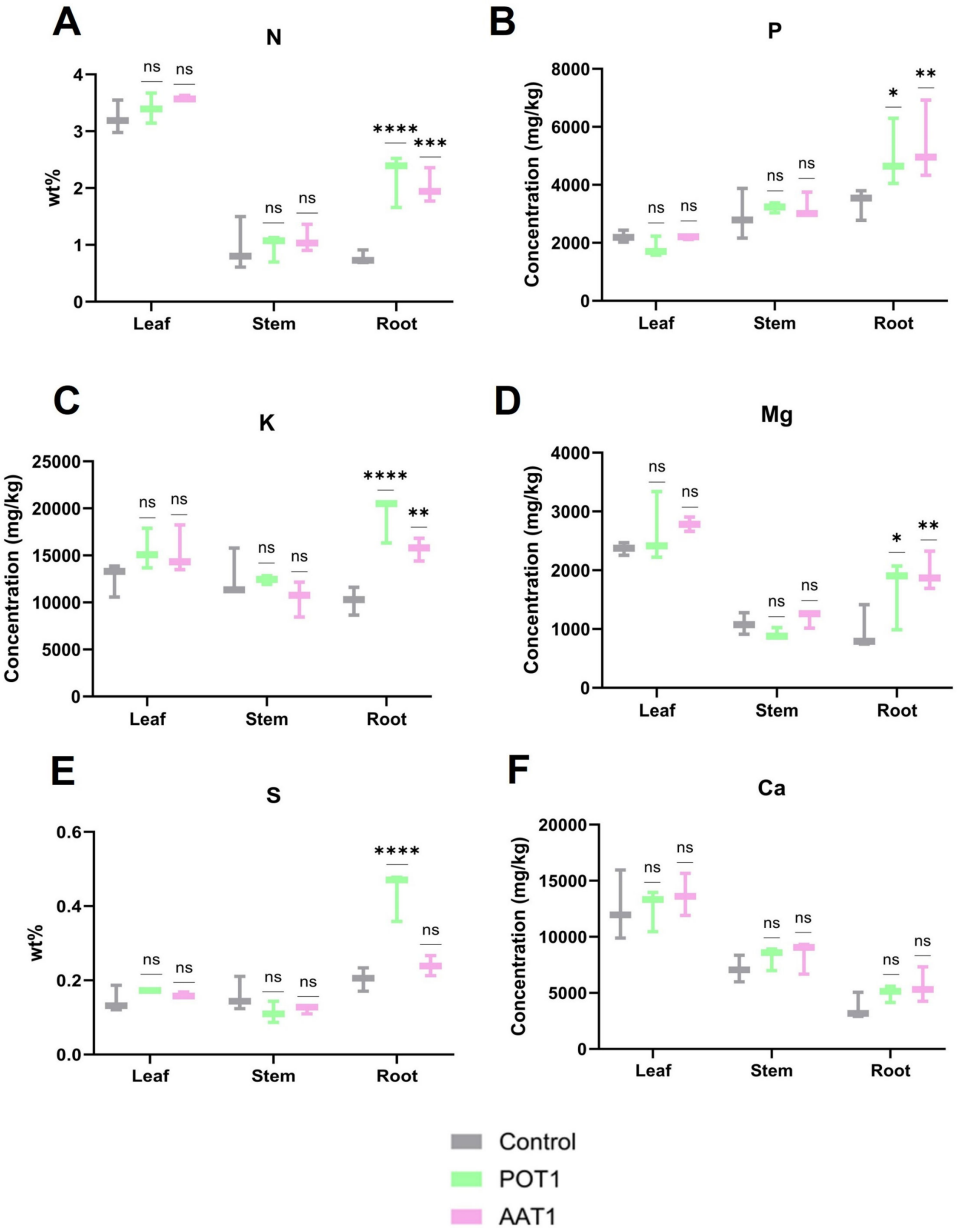
Elemental profiling of sengon treated with *P. olsonii* TLL1 (POT1) and *A. aculeatus* TLL1 (AAT1). Quantitative analysis of the macronutrients (**A**) nitrogen (N), (**B**) phosphorus (P), (**C**) potassium (K), (**D**) magnesium (Mg), (**E**) sulfur (S), and (**F**) calcium (Ca) in the leaf, stem, and root of the treated and uninoculated plants is shown. Data are presented as mean ± SD, with three biological replicates. Statistical significance was determined using two-way ANOVA, with significance levels indicated by * *P* < 0.05, ** *P* < 0.01, *** *P* < 0.001, **** *P* < 0.0001; ns, non-significant.

Nutrient analysis revealed a significant increase in root macronutrient levels under POT1 and AAT1 treatments, with nitrogen (N) increasing by approximately 2.5-fold, phosphorus (P) and potassium (K) by about 1.5-fold each, and magnesium (Mg) by nearly 1.75-fold (Fig. 4A through D and Table S3). In addition, sulfur (S) was significantly elevated only in POT1-treated plants, while calcium (Ca) did not show any variation in both treatments (Fig. 4E and F and Table S3). In leaf and stem samples, no significant difference in N, P, K, Mg, S, and Ca was detected upon POT1 and AAT1 inoculation. On the other hand, carbon (C) was significantly increased in both leaf and stem, and hydrogen (H) only in the stem of AAT1-inoculated plants. In POT1-inoculated plants, C was increased only in the leaf, while H did not show any difference compared with control (Fig. S7 and Table S3). Micronutrient levels of zinc (Zn), copper (Cu), and cobalt (Co) were significantly elevated in the roots upon treatment by POT1 and AAT1. In the stem, boron (B) showed significantly higher levels, whereas molybdenum (Mo) was lowered in both POT1- and AAT1-inoculated plants. The elements, such as sodium (Na) and manganese (Mn), did not differ in the treated plants compared with the control (Fig. S8 and Table S3).

### Metabolomic profiles of sengon in response to fungal colonization

To comprehensively explore the metabolic changes in sengon in response to fungal colonization, untargeted metabolomics was performed on the leaves of sengon plants treated with POT1 (SP) and AAT1 (SA) as well as uninoculated control plants (SC). A total of 1,694 and 2,819 metabolites were identified through metabolite profiling in negative and positive ion modes. Classification of these metabolites into chemical classes revealed that the fungal inoculation affected the major classes, including lipids, organoheterocyclic compounds, organic acids and derivatives, and phenylpropanoids and polyketides. The pie chart showing the detailed metabolite classification is given in Fig. S9.

### Differential metabolite analysis

**Fig 5:**
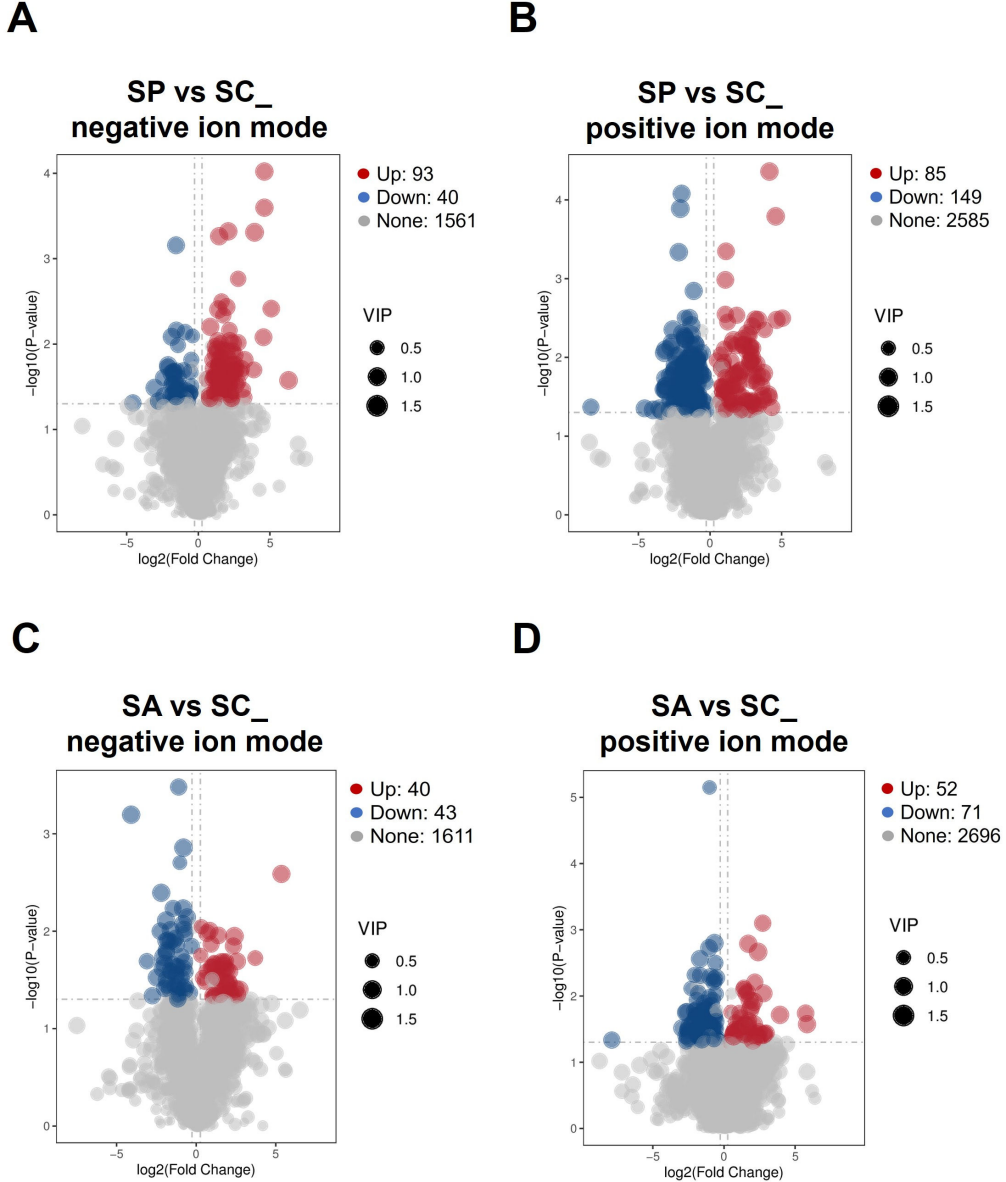
Volcano plot of differential metabolites in sengon upon colonization by the fungal isolates, *P. olsonii* TLL1 (POT1) and *A. aculeatus* TLL1 (AAT1). Volcano plots representing the differential metabolites in the pairwise combinations, SP vs SC (POT1 vs control) (**A**) in negative ion mode and (**B**) positive ion mode; and SA vs SC (AAT1 vs control) (**C**) in negative ion mode and (**D**) positive ion mode in are shown. Metabolites exhibiting a fold change greater than 2 and less than 0.5 with a *P* value of <0.05 and VIP > 1.0 were considered significantly differentially expressed for upregulated and downregulated metabolites, respectively. Each dot represents an identified metabolite, and the dots in red, blue, and gray represent up, down, and insignificant differential metabolites, respectively.

**Fig 6:**
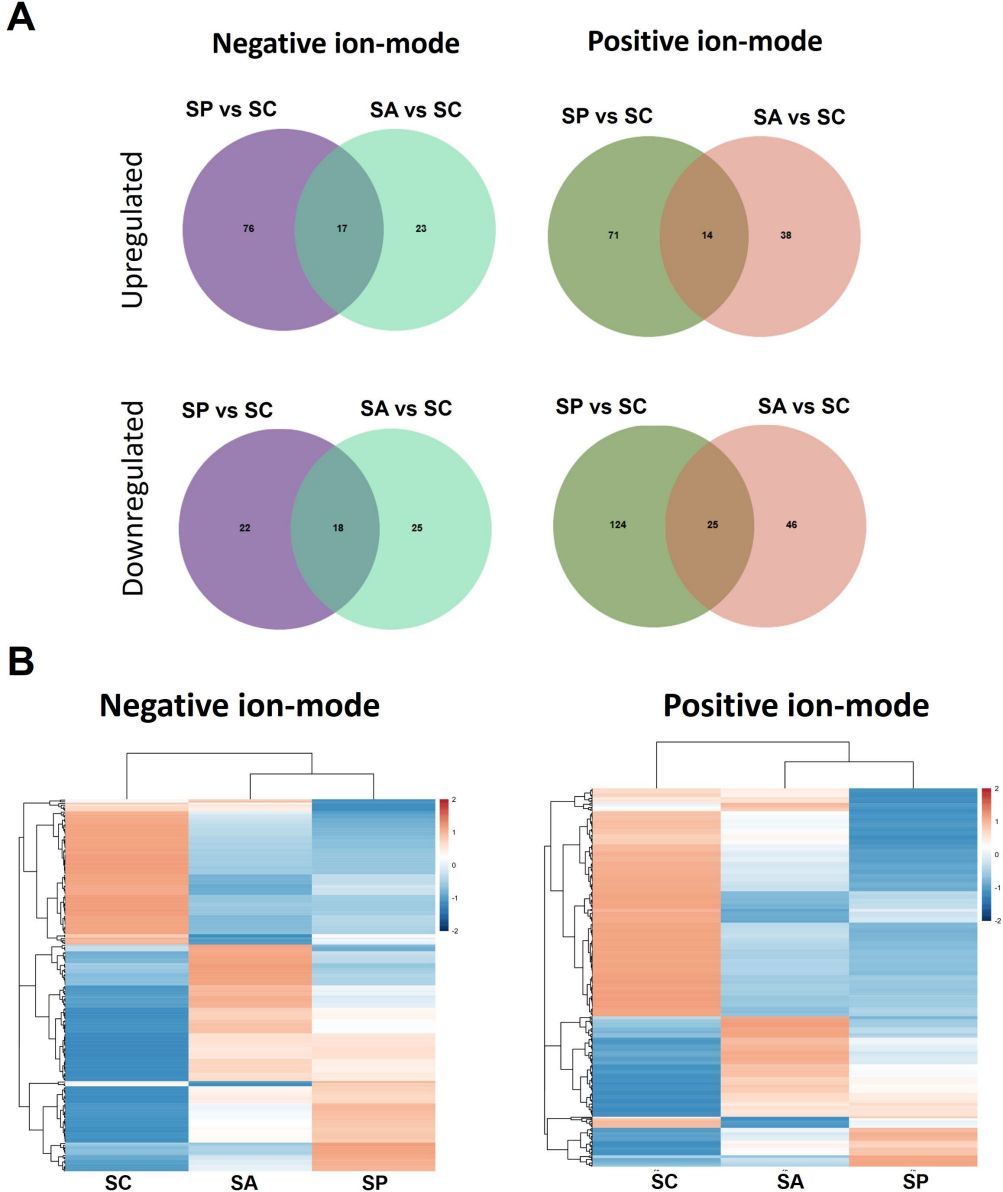
Venn diagrams and clustered heatmaps of differential metabolites in sengon colonized by *P. olsonii* TLL1 (POT1) and *A. aculeatus* TLL1 (AAT1). (**A**) Venn diagrams were plotted to show the overlapping and unique upregulated and downregulated differential metabolites among the comparison groups, SP vs SC (POT1 vs control) and SA vs SC (AAT1 vs control), in negative and positive ion modes. (**B**) Clustered heatmaps of differential metabolites in negative and positive ion modes were plotted to visualize the pattern in metabolite expression among the samples, SC (control), SA (AAT1), and SP (POT1). Each bar represents the abundance of each metabolite, and red and blue colors represent upregulated and downregulated metabolites, respectively.

**Fig 7:**
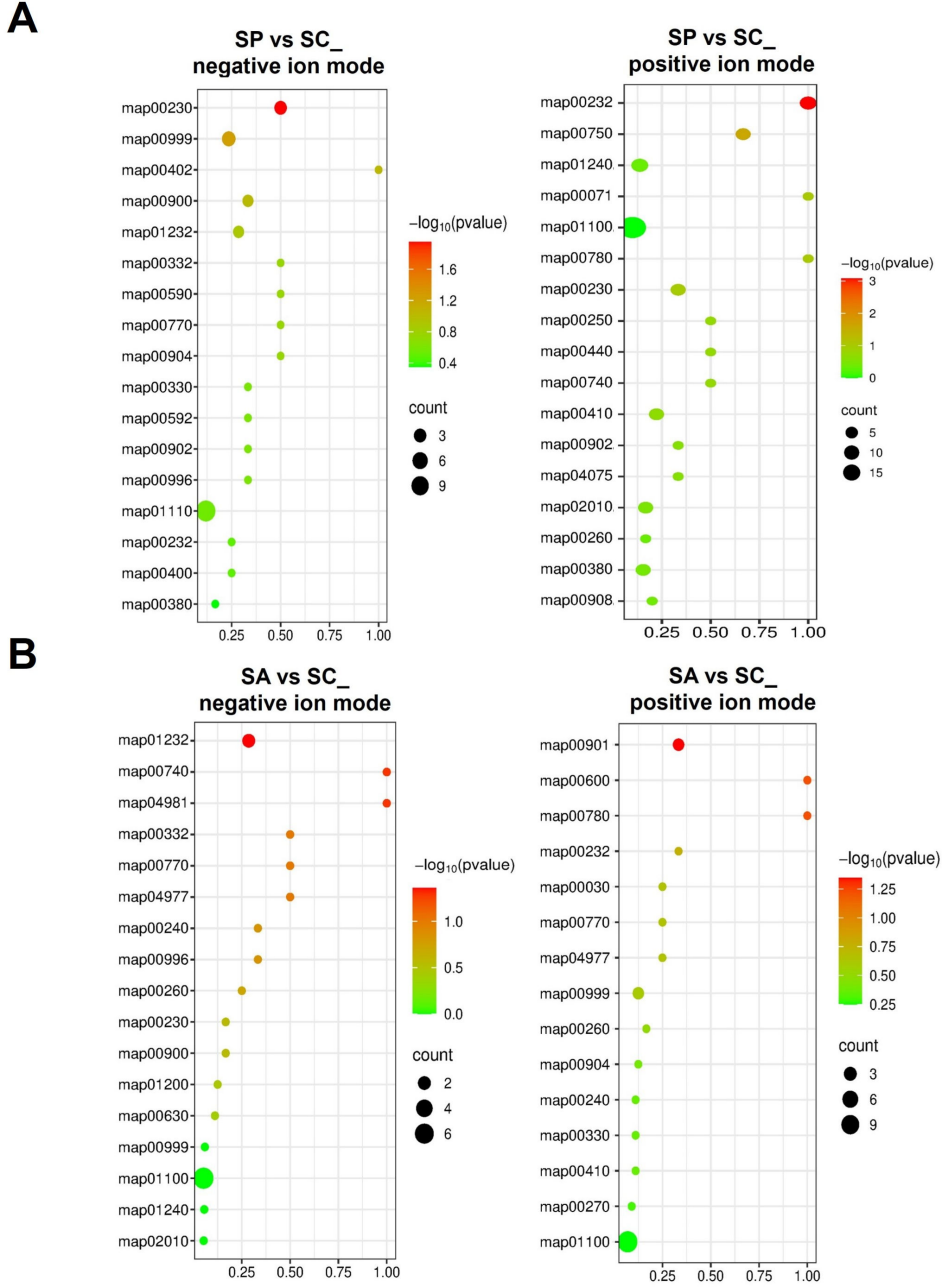
Kyoto Encyclopedia of Genes and Genomes (KEGG) enrichment pathways analysis of altered metabolites in sengon treated with *P. olsonii* TLL1 (POT1) and *A. aculeatus* TLL1 (AAT1). (**A**) Enrichment pathways of metabolites in negative and positive ion modes for the pairwise comparison, SP vs SC (POT1 vs control), showing the enriched metabolites following POT1 inoculation, and (**B**) for the pairwise comparison, SA vs SC (AAT1 vs control), showing the enriched metabolites following AAT1 inoculation. The *x*-axis represents the enrichment factor, and the *y*-axis represents the pathway ID. Bubble size represents the number of enriched metabolites in the pathway, and bubble color change from green to red indicates greater statistical significance.

To identify the impact of fungal colonization on sengon, the metabolomic profile of leaves from treated plants was compared against that of the uninoculated control. The differential metabolite screening was performed between two pairwise comparison groups, SP vs SC and SA vs SC. The comparative metabolic profile of sengon revealed notable differences in effects by the two fungal isolates, POT1 and AAT1. In POT1-treated plants, 133 (93 upregulated and 40 downregulated) and 234 (85 upregulated and 149 downregulated) metabolites were significantly regulated under negative and positive modes, respectively. Likewise, in AAT1-treated plants, 83 (40 upregulated and 43 downregulated) and 123 (52 upregulated and 71 downregulated) metabolites were significantly regulated under negative and positive modes, respectively (File S1). Volcano plots representing the overall distribution of differential metabolites are given in Fig. 5. Through multigroup comparisons, overlapping and unique metabolites between SP vs SC and SA vs SC were identified. Under negative ion mode, 17 upregulated and 18 downregulated metabolites were commonly expressed, while under positive ion mode, 14 upregulated and 25 downregulated metabolites were detected under the same category (File S2). Compared with overlapping metabolites, the number of unique metabolites was higher in both groups under negative and positive ion modes. The Venn diagrams displaying the distribution of unique and common metabolites among the upregulated and downregulated are given in Fig. 6A. The control and fungal treatment groups were clearly distinguished from one another by clustering heatmaps in both negative and positive ion modes (Fig. 6B). The pathways regulated under fungal colonization were further explored by KEGG enrichment analysis (Fig. 7 and File S3). The pathways, namely metabolic pathways (map01100), biosynthesis of plant secondary metabolites (map00999), vitamin digestion and absorption (map04977), glycine, serine, and threonine metabolism (map00260), and nucleotide metabolism (map01232) were the major pathways enriched in both POT1- and AAT1-treated plants.

### Metabolites related to plant growth and photosynthesis

**Fig 8:**
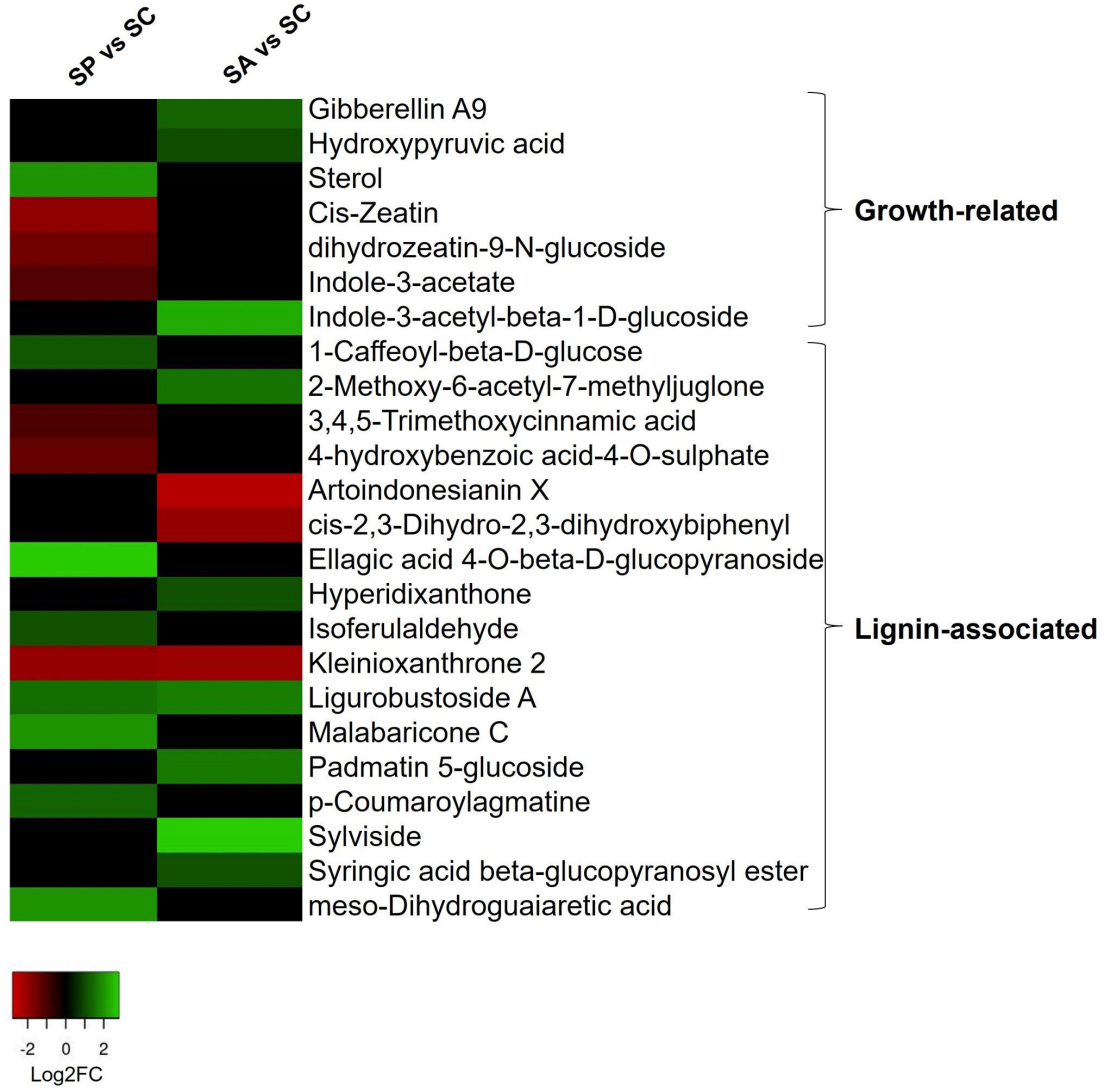
Heatmap showing growth- and lignification-related differential metabolites in sengon treated with *P. olsonii* TLL1 (POT1) and *A. aculeatus* TLL1 (AAT1). Differential metabolites in the pairwise comparisons: SP vs SC (POT1 vs control) and SA vs SC (AAT1 vs control) are shown. The metabolites exhibiting a fold change greater than two and less than 0.5 with a value of <0.05 and VIP >1.0 were considered significantly differentially expressed for upregulated and downregulated metabolites, respectively. The heat maps were constructed using the log2 fold change values, and green and red colors represent upregulated and downregulated metabolites, respectively.

To assess which of the metabolites are responsible for observed differences in growth that correlated with POT1 and AAT1 inoculation, growth- and photosynthesis-associated metabolites were identified (Fig. 8 and File S4). In POT1-treated plants, growth-related metabolites, such as cis-zeatin, dihydrozeatin-9-N-glucoside, and indole-3-acetate were decreased, while sterol essential for membrane synthesis and wood formation was upregulated. Upon AAT1 inoculation, hormone-related gibberellin A9 and indole-3-acetyl-beta-1-D-glucoside were prominently accumulated, correlating with the growth promotion in AAT1-treated plants. Additionally, an intermediate metabolite of the photorespiration pathway involved in photosynthesis-supporting metabolism, hydroxypyruvic acid, was accumulated in AAT1-treated plant, indicating active photosynthetic metabolism.

### Metabolites linked to lignin synthesis

Figure 8 summarizes differentially abundant metabolites associated with lignin-related secondary metabolism in sengon leaves following fungal colonization. Several phenylpropanoid- and lignan-related metabolites, including isoferulaldehyde, syringic acid β-glucopyranosyl ester, p-coumaroylagmatine, meso-dihydroguaiaretic acid, and 1-caffeoyl-β-D-glucose, were significantly altered in fungal-treated plants compared with uninoculated controls (File S4). Fungal colonization modulated the abundance of multiple metabolites that support phenylpropanoid flux toward lignin- and lignan-associated pathways, which are closely linked to secondary cell wall-related metabolic processes in woody plants. In POT1-treated plants, isoferulaldehyde, 1-caffeoyl-β-D-glucose, ellagic acid 4-O-β-D-glucopyranoside, meso-dihydroguaiaretic acid, and p-coumaroylagmatine were elevated, whereas AAT1-treated plants showed increased levels of syringic acid β-glucopyranosyl ester, ligurobustoside A, padmatin 5-glucoside, and sylviside.

### Metabolites associated with stress- and defense-related secondary metabolism

**Fig 9:**
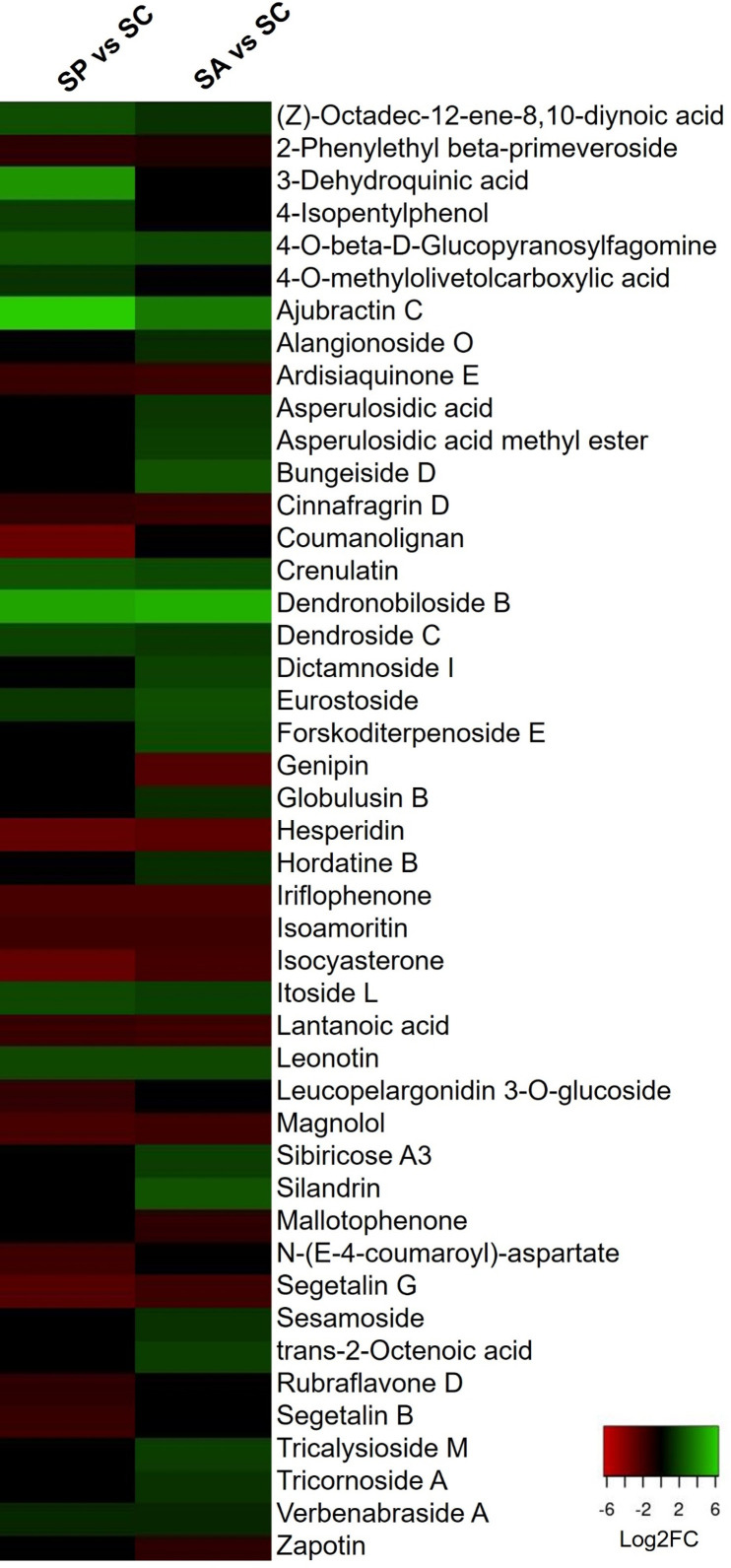
Heatmap showing defense-related differential metabolites in sengon treated with *P. olsonii* TLL1 (POT1) and *A. aculeatus* TLL1 (AAT1). Differential metabolites in the pairwise comparisons: SP vs SC (POT1 vs. control) and SA vs SC (AAT1 vs control) are shown. The metabolites exhibiting a fold change greater than two and less than 0.5 with a value of <0.05 and VIP > 1.0 were considered significantly differentially expressed for upregulated and downregulated metabolites, respectively. The heat maps were constructed using the log2 fold change values, and green and red color represent upregulated and downregulated metabolites, respectively.

As shown in Fig. 9, several metabolic changes observed in this study are associated with stress- and defense-related secondary metabolism, indicating chemical reprogramming of sengon in response to fungal colonization (File S4). In both POT1- and AAT1-treated plants, terpenoids and terpenoid glycosides constituted a prominent class of differentially abundant metabolites. Notably, asperulosidic acid, asperulosidic acid methyl ester, dictamnoside I, tricalysioside M, and globulusin B were specifically elevated in AAT1-treated plants, whereas other terpene glycosides, including verbenabraside A, dendroside C, dendronobiloside B, and eurostoside, showed increased abundance in both POT1- and AAT1-inoculated plants. These terpenoid-derived compounds are commonly associated with inducible chemical responses and stress-adaptive metabolism in plants.

In addition, POT1 treatment resulted in higher levels of 4-O-methylolivetolcarboxylic acid and 4-isopentylphenol, both phenolic secondary metabolites, along with 3-dehydroquinic acid, a key intermediate of the shikimate pathway. The accumulation of these metabolites suggests enhanced flux through aromatic amino acid–derived secondary metabolic pathways. Other metabolites implicated in stress-associated chemical responses included (Z)-octadec-12-ene-8,10-diynoic acid, a polyacetylenic fatty acid that was elevated in both POT1- and AAT1-treated plants.

Conversely, several flavonoids, including hesperidin, isoamoritin, rubraflavone D, zapotin, and leucopelargonidin 3-O-glucoside, were reduced following fungal colonization. This reduction suggests a potential reallocation of metabolic precursors away from flavonoid biosynthesis toward alternative secondary metabolic pathways, rather than uniform activation of classical defense responses.

## DISCUSSION

The growing demand for environmentally sustainable alternatives to chemical fertilizers and pesticides has intensified interest in beneficial plant–microbe interactions for forestry and plantation systems. In this study, we demonstrate that two fungal isolates, *P. olsonii* TLL1 (POT1) and *A. aculeatus* TLL1 (AAT1), exhibit pronounced functional divergence in their interactions with sengon (*F. moluccana*), resulting in distinct growth, nutritional, and metabolic outcomes. Although both fungi established non-pathogenic associations with the host, only AAT1 consistently translated fungal colonization into enhanced biomass accumulation, whereas POT1 primarily induced metabolic reprogramming without measurable growth promotion. These findings highlight that fungal colonization alone is insufficient for growth enhancement; instead, functional compatibility between fungal traits and host metabolic priorities is critical.

### Functional divergence between fungal isolates in sengon

Both POT1 and AAT1 colonized sengon roots without eliciting visible disease symptoms, indicating compatible interactions. However, growth responses diverged markedly at later developmental stages, with AAT1-treated plants showing significant increases in plant height, leaf number, and shoot and root biomass, while POT1-treated plants exhibited only marginal or transient growth effects. This functional divergence indicates that growth promotion is not a default consequence of fungal colonization but a strain-specific outcome contingent on the compatibility between microbial traits—particularly hormone-related metabolite production and colonization architecture—and the host’s metabolic and developmental context. Similar isolate-specific outcomes have been reported in other systems, emphasizing that plant growth-promoting activity is highly context- and host-dependent (6, 7). Our findings extend this concept to a fast-growing tropical tree species, underscoring the need to evaluate microbial inoculants directly in woody hosts rather than extrapolating from herbaceous models. An important avenue for future work is the quantitative assessment of fungal colonization levels within roots, for example, by real-time qPCR using species-specific primers, which would enable direct correlation between colonization intensity and growth or metabolic outcomes. Such data would complement the qualitative colonization evidence presented here and strengthen mechanistic interpretations of the observed host responses.

### Coupling of nutrient acquisition and metabolic reprogramming

Elemental profiling revealed that fungal colonization substantially enhanced the accumulation of multiple macro- and micronutrients in sengon roots, including nitrogen, phosphorus, magnesium, and zinc. These nutrients are fundamental drivers of both primary metabolism and secondary metabolic investment (25). Although nutrient and metabolomic analyses were conducted on different tissues, the coordinated patterns observed suggest a functional coupling between belowground nutrient acquisition and aboveground metabolic allocation. Enhanced nutrient availability likely alleviated resource limitations, allowing sengon to maintain growth while reallocating carbon toward secondary metabolic pathways, particularly phenylpropanoid-, lignin-, and terpenoid-associated metabolism.

Such nutrient-supported metabolic reprogramming is especially relevant for woody plants, which rely on sustained investment in secondary metabolism for long-term structural reinforcement, defense, and biomass accumulation (14, 15). Rather than reflecting a classical growth–defense trade-off, the observed metabolic shifts are consistent with a strategy in which improved nutritional status enables simultaneous support of growth and inducible defense.

### POT1 induces defense- and lignification-oriented metabolic reprogramming

Although POT1 did not promote biomass accumulation at harvest, untargeted metabolomics revealed pronounced redirection of host metabolism toward structural reinforcement and defense-associated pathways. POT1-treated plants accumulated metabolites linked to hydroxycinnamic acid amide formation, lignan biosynthesis, and monolignol metabolism, including p-coumaroylagmatine, meso-dihydroguaiaretic acid, isoferulaldehyde, and 1-caffeoyl-β-D-glucose. In woody species, these metabolite classes are closely associated with secondary cell wall formation, xylem differentiation, and mechanical strength (22, 23), suggesting that POT1-treated plants preferentially invest in wood-quality and defense-related traits rather than rapid biomass expansion.

The induction of p-coumaroylagmatine, a hydroxycinnamic acid amide known to enhance antimicrobial resistance and cell wall reinforcement (26–30), together with increased shikimate pathway activity indicated by elevated 3-dehydroquinic acid, points to activation of phenylpropanoid-based defense metabolism. In addition, the accumulation of iridoids and polyacetylenic fatty acids, which possess antimicrobial and anti-herbivore properties (31–34), further supports a defense-primed metabolic state. Notably, several flavonoids were reduced upon POT1 treatment, suggesting a reallocation of metabolic precursors away from flavonoid biosynthesis toward lignin-, HCAA-, and iridoid-related pathways rather than uniform activation of all defense branches.

Importantly, the lack of growth promotion by POT1 in sengon does not imply a general limitation of this isolate. POT1 has previously been shown to enhance growth and stress tolerance in other plant species under specific environmental constraints, such as phosphate deficiency (8). Together, these observations indicate that POT1 acts as a metabolic modulator that prioritizes structural reinforcement and defense priming, which may confer adaptive advantages under stress-prone or pathogen-rich environments.

### AAT1 integrates nutrient acquisition with growth and secondary metabolism

In contrast to POT1, AAT1 established a highly compatible interaction with sengon, effectively coupling nutrient acquisition, hormonal regulation, and metabolic reprogramming with robust growth promotion. AAT1-treated plants exhibited increased accumulation of growth-associated phytohormone metabolites, including auxin- and gibberellin-related compounds, which are known regulators of cambial activity, vascular differentiation, and shoot elongation in woody plants (35, 36). These hormonal shifts are consistent with the enhanced biomass accumulation observed at harvest.

At the same time, AAT1 also modulated secondary metabolism, promoting the accumulation of syringyl-associated lignin precursors, such as syringic acid β-glucopyranosyl ester and defense-related iridoid glycosides. Syringyl-rich lignin is associated with efficient lignification and improved cell wall flexibility, which may support both mechanical stability and sustained growth in fast-growing tree species (37). The concurrent enhancement of iridoids indicates that growth promotion by AAT1 does not come at the expense of chemical defense capacity.

The ability of AAT1 to simultaneously enhance nutrient status, growth-related hormone metabolism, and selective secondary metabolism suggests a coordinated metabolic strategy rather than a growth-defense trade-off. This integration likely reflects specific functional traits of *A. aculeatus*, including organic acid secretion, phosphate solubilization, and production of hormone-like compounds (38, 39), which together create a permissive nutritional and signaling environment for host metabolic plasticity.

### Defense priming rather than constitutive defense activation

Across both fungal treatments, the overall metabolic signature is indicative of defense priming rather than constitutive defense activation. Defense priming enables plants to remain poised for rapid and energy-efficient responses upon biotic challenge while minimizing fitness costs under non-stress conditions (40, 41). The coordinated induction of lignin-related phenolics, hydroxycinnamic acid amides, iridoids, and lipid-derived defense compounds, together with repression of selected flavonoids, supports the notion that fungal colonization reconfigures sengon metabolism toward an inducible, rather than permanently activated, defense state. Such a strategy is particularly advantageous for long-lived woody species that must balance growth, structural investment, and resilience over extended life spans.

### Plant growth-promoting traits of indoor dust-associated fungi

The observation that both beneficial fungal isolates in this study originated from house dust raises the question of why environmental (non-rhizospheric) fungi may harbor plant growth-promoting traits. We propose that selective pressures in indoor environments—including nutrient scarcity and competitive microbial interactions—favor the maintenance of metabolic capabilities, such as organic acid secretion, phosphate solubilization, and secondary metabolite production, that overlap functionally with traits associated with rhizosphere competence. *Aspergillus* and *Penicillium* species are known producers of auxin- and gibberellin-related compounds and hydrolyzing enzymes (38, 39), and may therefore represent an untapped bioprospecting resource for plant growth-promoting microorganisms. Future comparative studies between environmental and rhizospheric isolates of the same genus would be valuable to test this hypothesis.

### Conclusions and implications for sustainable forestry

In conclusion, our study demonstrates that dust-borne beneficial fungi exert isolate-specific effects on sengon growth and metabolism. *A. aculeatus* TLL1 (AAT1) emerges as a functionally superior growth-promoting fungus, effectively integrating nutrient acquisition, hormonal regulation, and metabolic reprogramming to support both biomass accumulation and metabolic resilience. In contrast, *P. olsonii* TLL1 (POT1) primarily induces defense- and lignification-oriented metabolic shifts without enhancing growth under the conditions tested. These findings highlight the importance of host-fungus functional compatibility and underscore that effective bioinoculant selection for forestry applications must consider not only colonization capacity but also the alignment of microbial traits with host metabolic and developmental priorities. By providing a metabolome-scale view of fungus-tree interactions, this work establishes a framework for rational deployment of beneficial fungi in sustainable forestry and plantation management systems.

## MATERIALS AND METHODS

### Fungal and plant material

The present study was conducted using two fungal isolates, POT1 (*P. olsonii* TLL1) (8) and AAT1 (*A. aculeatus* TLL1), isolated from indoor dust samples. The fungal isolates were cultured on Malt Extract Agar (MEA; pH 5.6) at 28 ± 2°C for 7 days. Fungal spore suspensions prepared in sterile distilled water (10^8^ spores/mL) were inoculated into Malt Extract Broth (MEB; pH 5.6), and mycelia were harvested after 7 days at 28 ± 2°C.

Commercially available seeds of Sengon (*F. moluccana*) were surface sterilized with 70% ethanol and 5% hydrogen peroxide for 2 and 10 min, respectively, germinated on Murashige and Skoog (MS) medium with B5 vitamins (Duchefa, Haarlem, Netherlands) (pH 6.0) and maintained at 25°C under sterile *in vitro* conditions. One-month-old seedlings were acclimatized under greenhouse conditions before planting in soil for the experiments.

### Identification and genotyping of fungus

The genotyping of POT1 has already been described in our previous study by (8). For AAT1, a total of five fungus barcoding genes; *ITS, LSU, TEF1α, BenA*, and *MCM7* were amplified using 1 µL of DNA in a standard PCR amplification of 35 cycles with the Phusion High-Fidelity DNA Polymerase kit (ThermoFisher Scientific, Waltham, MA, USA). Detailed primer sequences used are listed in Table S4. PCR products were cleaned with ExoSap-ITTM (Applied Biosystems, Waltham, MA, USA), and sequencing reactions were performed using BigDye Terminator v3.1 (Applied Biosystems, Waltham, MA, USA). Sequences were determined using Sanger’s sequencing and analyzed with Benchling (Benchling Inc.). Obtained sequences were blasted on the GenBank nucleotide database (http://blast.ncbi.nlm.nih.gov/) to identify similar genes. The sequences were assembled and aligned with our obtained sequences using Clustal W and trimmed in MEGA v.7.0. Maximum-likelihood (ML) trees were generated using the trimmed aligned sequences. The Tamura-Nei model was used, and ML analyses were done by calculating the initial tree with Bio-Neighbour-Joining (BioNJ). Node support was calculated by bootstrapping with 1,000 replicates (42).

### Experimental design

For plant growth experiments, fungal mycelia resuspended in distilled water were inoculated in a potting mixture (BVB substrate: soil: sand 3:1:1) and pre-cultured for 7 days. For the control treatment, uninoculated soil was used. Subsequently, 1-month-old sengon seedlings were transplanted into the treated and control soil and grown under greenhouse conditions. Particularly, six plants were maintained for each treatment and designated as (i) control, (ii) POT1, and (iii) AAT1. The growth parameters, plant height and number of leaves, were recorded at monthly intervals after treatment. The plants were harvested after 6 months, and growth parameters, namely plant height, number of leaves, shoot weight, leaf weight and root weight, were measured.

### Estimation of total chlorophyll content

Total chlorophyll content in leaves was analyzed using a chlorophyll meter (SPAD-502, Minolta, Japan). All six biological replicates of the three treatments were taken to estimate the chlorophyll content. The measurements were collected on the leaf lamina by avoiding the midrib, and a minimum of 10 values were recorded for each plant for SPAD measurements.

### Detection of fungal colonization by microscopy

For detection of fungal colonization of sengon, sprouted seeds were transplanted onto MS media plates pre-cultured with 100 µL of fungal spores (10^8^ spores/mL) and grown for 2 weeks. Seedlings grown on uninoculated plates served as control. The roots excised from treated and control plants were stained using Wheat Germ Agglutinin-Alexa Fluor 488 conjugate (WGA-AF488) and propidium iodide (PI) (43), and confocal analysis was performed using FV3000 confocal laser scanning microscope (Olympus, Tokyo, Japan). Excitation/detection wavelength was at 488/500–540 nm for WGA-AF488 and at 560/580–630 nm for PI.

### Assessment of mineral composition by CHNS and ICP analysis

For elemental profiling, leaf, stem, and root samples excised from 6-month-old plants were used. The samples were dried at 60°C for 3–5 days, ground into fine powders, and the powdered samples in three biological replicates were used for analysis. Elements, such as carbon, hydrogen, nitrogen, and sulfur, were assessed using Vario El cube (Elementar, Hanau, Germany). A typical weight of 2 mg of the samples was pyrolyzed in helium (He) atmosphere, and the C/H/N/S elements in pyrolyzed gases were analyzed using respective detectors. For analysis of other elements, namely phosphorus, molybdenum, iron, potassium, boron, calcium, cobalt, copper, magnesium, manganese, and sodium, and zinc, ~100 mg of the powdered samples was digested according to the methods previously described (44). The digested samples were cooled to room temperature in a fume hood, diluted to 10 mL with deionized water, and analyzed using Agilent 720 Inductively Coupled Plasma-Optical Emission Spectrometry (ICP-OES) system (Agilent Technologies, Santa Clara, CA, USA) with a detection limit of 0.02 ppm and Agilent 7700s Inductively Coupled Plasma-Mass Spectrometry (ICP-MS) system (Agilent Technologies, Santa Clara, CA, USA) with a detection limit of 0.001 ppm.

### Metabolomic analysis

For untargeted metabolomics, three replicates of each treatment, control (SC), POT1 (SP), and AAT1 (SA), were processed. Leaves collected from 6-month-old plants of each treatment were immediately frozen in liquid nitrogen and stored at −80°C. The metabolites were extracted from 100 mg of finely ground leaf tissues with pre-chilled 80% methanol, centrifuged at 15,000 × *g* at 4°C for 20 min, and the supernatant was used for analysis. For metabolomics analysis, ultra-high performance liquid chromatography (UHPLC)-MS/MS analyses were performed using a Vanquish UHPLC system (ThermoFisher, Bremen, Germany) coupled with an Orbitrap Q Exactive HF-X mass spectrometer (ThermoFisher, Bremen, Germany). Chromatographic separation was carried out on a Hypersil Gold column (100 × 2.1 mm, 1.9 μm) with a flow rate set at 0.2 mL/min. The eluents for the positive and negative polarity modes were eluent A (0.1% formic acid in water) and eluent B (methanol). The solvent gradient was set as follows: 1.5 min, 2% B; 3 min, 2%–85% B; 10 min, 85%–100% B; 10.1 min, 100%–2% B; 12 min, 2% B. The Q Exactive HF mass spectrometer was operated in positive/negative polarity mode with spray voltage of 3.5 kV, sheath gas flow rate of 35 psi, capillary temperature of 320°C, auxiliary gas flow rate of 10 L/min, S-lens RF level of 60, and auxiliary gas heater temperature of 350°C.

The raw data files from UHPLC-MS/MS were processed by XCMS software for performing peak detection, alignment, and quantitation of each metabolite, followed by metabolite identification using the NovoMetDB database (Novogene, Beijing, China). The metabolite data collected in positive and negative ion modes were annotated using the KEGG database (https://www.genome.jp/kegg/pathway.html), HMDB database (https://hmdb.ca/metabolites), and LIPID MAPS database (http://www.lipidmaps.org/). Differential metabolites of the pairwise comparisons (SP vs SC and SA vs SC) were screened based on the criteria: variable importance in projection (VIP) value ≥1, *P*-value ≤ 0.05, and fold change (FC) ≥2 or ≤0.5. Volcano plots and clustering heatmaps were plotted using R version 3.4.3 software. Further, KEGG enrichment analysis of differentially abundant metabolites was conducted, and the pathway was considered significantly enriched when the conditions of pathway component ratio (x/n) > background ratio (y/N) and Benjamini-Hochberg adjusted *P*-value < 0.05 were met.

### Statistical analysis

Statistical analysis was performed using GraphPad Prism software version 9.5 for Windows (GraphPad Software, San Diego, CA, USA). Assessment of data in replicates was conducted using two-tailed Student’s *t*-test and two-way ANOVA, and significant differences across treatments were designated as ***P < 0.001, **P < 0.01, and *P < 0.05.

## Data Availability

The sequences of the barcode markers of *A. aculeatus* (AAT1) used for construction of the phylogenetic trees are available in the GenBank database under the following accession numbers: PX953049 (*ITS*), PX953050 (*LSU*), PX975904 (*TEF1α*), PX975905 (*BenA*), and PX975906 (*MCM7*).

## SUPPLEMENTAL MATERIAL

Data S1 aem.00412-26-s0001.xlsx Differential expression analysis of metabolites in sengon.

Data S2 aem.00412-26-s0002.xlsx Differential metabolites of multiple comparison combinations.

Data S3 aem.00412-26-s0003.xlsx KEGG enrichment analysis of differential metabolites in sengon.

Data S4 aem.00412-26-s0004.xlsx Differential metabolites in sengon.

Supplemental figures aem.00412-26-s0005.docx Fig. S1 to S9.

Supplemental tables aem.00412-26-s0006.xlsx Tables S1 to S4.

ASM does not own the copyrights to Supplemental Material that may be linked to, or accessed through, an article. The authors have granted ASM a non-exclusive, world-wide license to publish the Supplemental Material files. Please contact the corresponding author directly for reuse.
