# Supplementary material for: Beneficial fungi reprogram tree metabolism toward growth and stress-associated pathways
Source: Appl Environ Microbiol. 2026 Jun 18;92(7):e00412-26. doi: 10.1128/aem.00412-26 (PMC13390345; doi:10.1128/aem.00412-26)
Supplement: Supplemental figures — Fig. S1 to S9. [file aem.00412-26-s0005.docx]

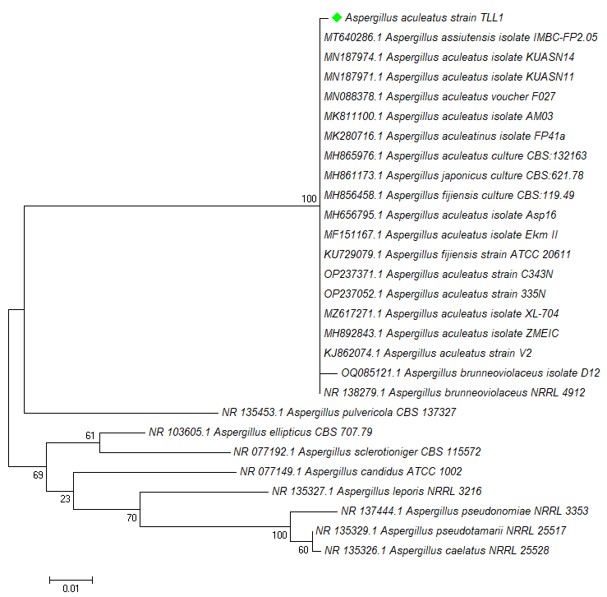


**FIG S1.** Maximum-likelihood tree of the plant growth promoting fungus, *A. aculeatus* TLL1 (AAT1) based on *ITS* sequences. Best-scoring Maximum Likelihood (ML) tree, constructed using Tamura-Nei model within MEGA version 7.0 based on partial internal transcribed spacer (*ITS*) sequences, showing the clustering of AAT1 with other *Aspergillus* strains. The bootstrap values of the ML analysis are presented at the nodes.


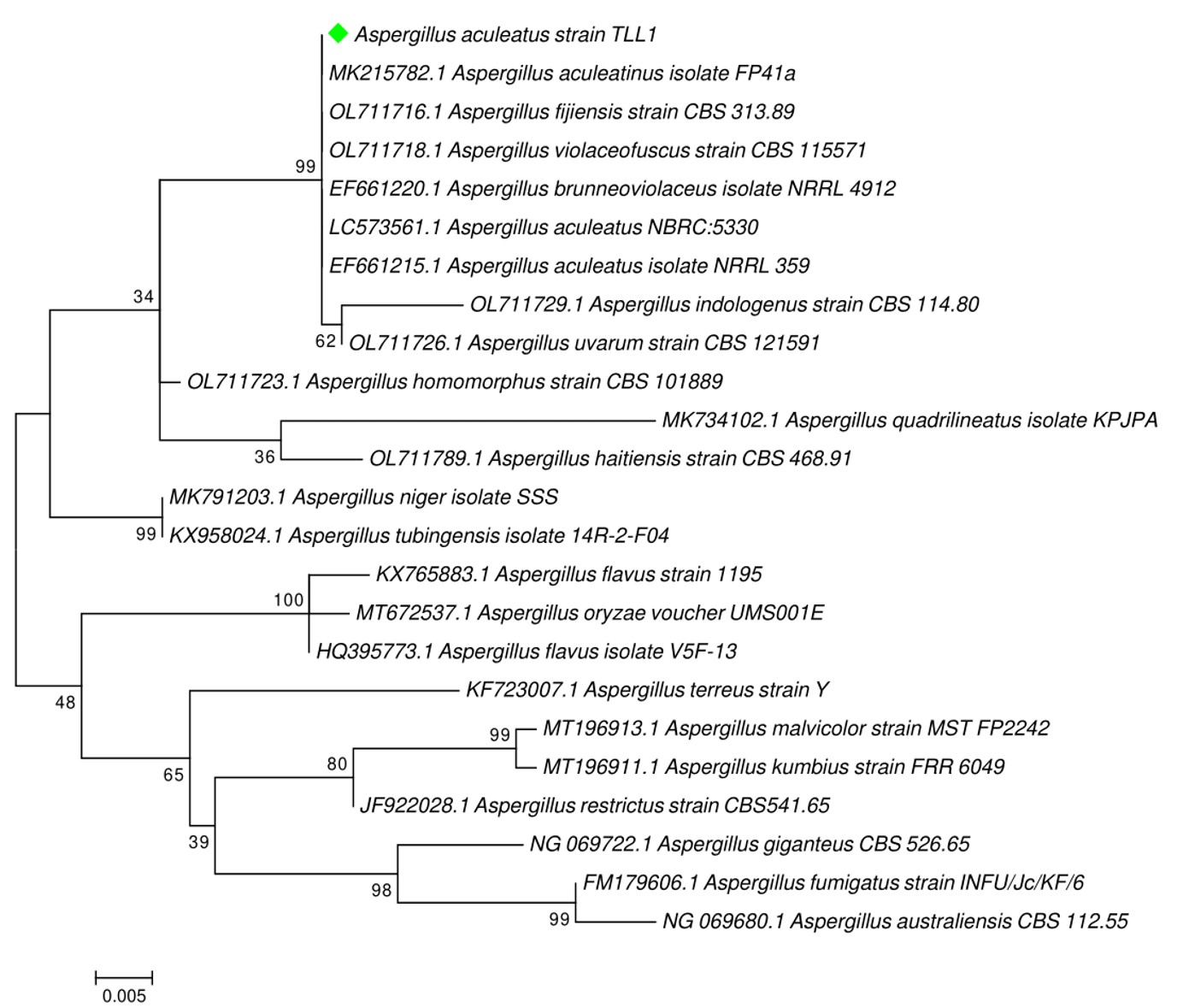


**FIG S2.** Maximum-likelihood tree of the plant growth promoting fungus, *A. aculeatus* TLL1 (AAT1) based on large ribosomal subunit (*LSU*) sequences. Best-scoring Maximum Likelihood (ML) tree, constructed using Tamura-Nei model within MEGA version 7.0 based on partial large ribosomal subunit (*LSU*) sequences, showing the clustering of AAT1 with other *Aspergillus* strains. The bootstrap values of the ML analysis are presented at the nodes.


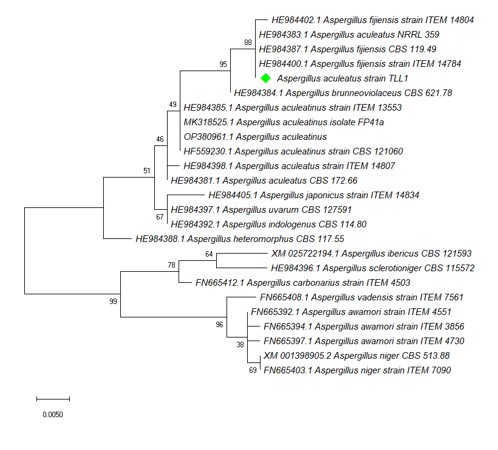


**FIG S3.** Maximum-likelihood tree of the plant growth promoting fungus, *A. aculeatus* TLL1 (AAT1) based on translation elongation factor-1-α (*TEF1-α*) sequences. Best-scoring Maximum Likelihood (ML) tree constructed using Tamura-Nei model within MEGA version 7.0 based on partial translation elongation factor-1-α (*TEF1-α*) sequences, showing the clustering of AAT1 with other *Aspergillus* strains. The bootstrap values of the ML analysis are presented at the nodes.


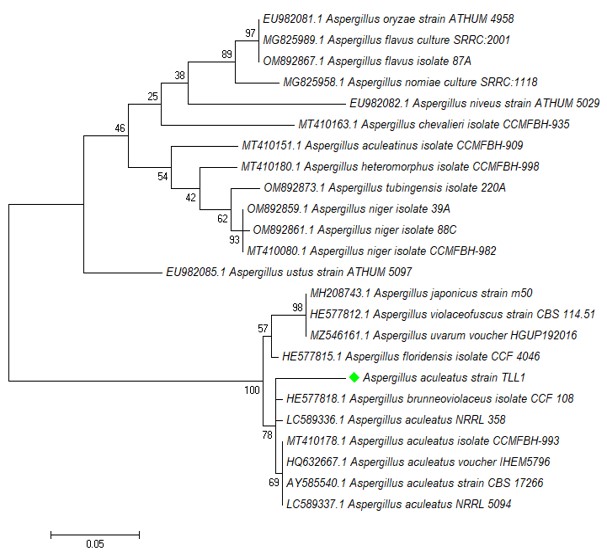


**FIG S4.** Maximum-likelihood tree of the plant growth promoting fungus, *A. aculeatus* TLL1 (AAT1) based on beta-tubulin (*BenA*) sequences. Best-scoring Maximum Likelihood (ML) tree, constructed using Tamura-Nei model within MEGA version 7.0 based on partial beta-tubulin (*BenA*) sequences, showing the clustering of AAT1 with other *Aspergillus* strains. The bootstrap values of the ML analysis are presented at the nodes.


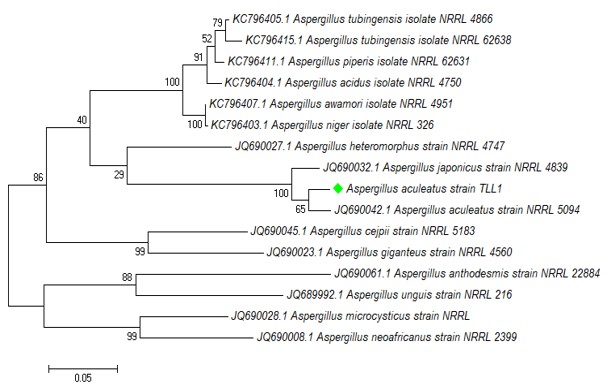


**FIG S5.** Maximum-likelihood tree of the plant growth promoting fungus, *A. aculeatus* TLL1 (AAT1) based on mini-chromosome maintenance protein (*MCM7*) sequences. Best-scoring Maximum Likelihood (ML) tree, constructed using Tamura-Nei model within MEGA version 7.0 based on partial Mini-chromosome maintenance protein (*MCM7*) sequences, showing the clustering of AAT1 with other *Aspergillus* strains. The bootstrap values of the ML analysis are presented at the nodes.


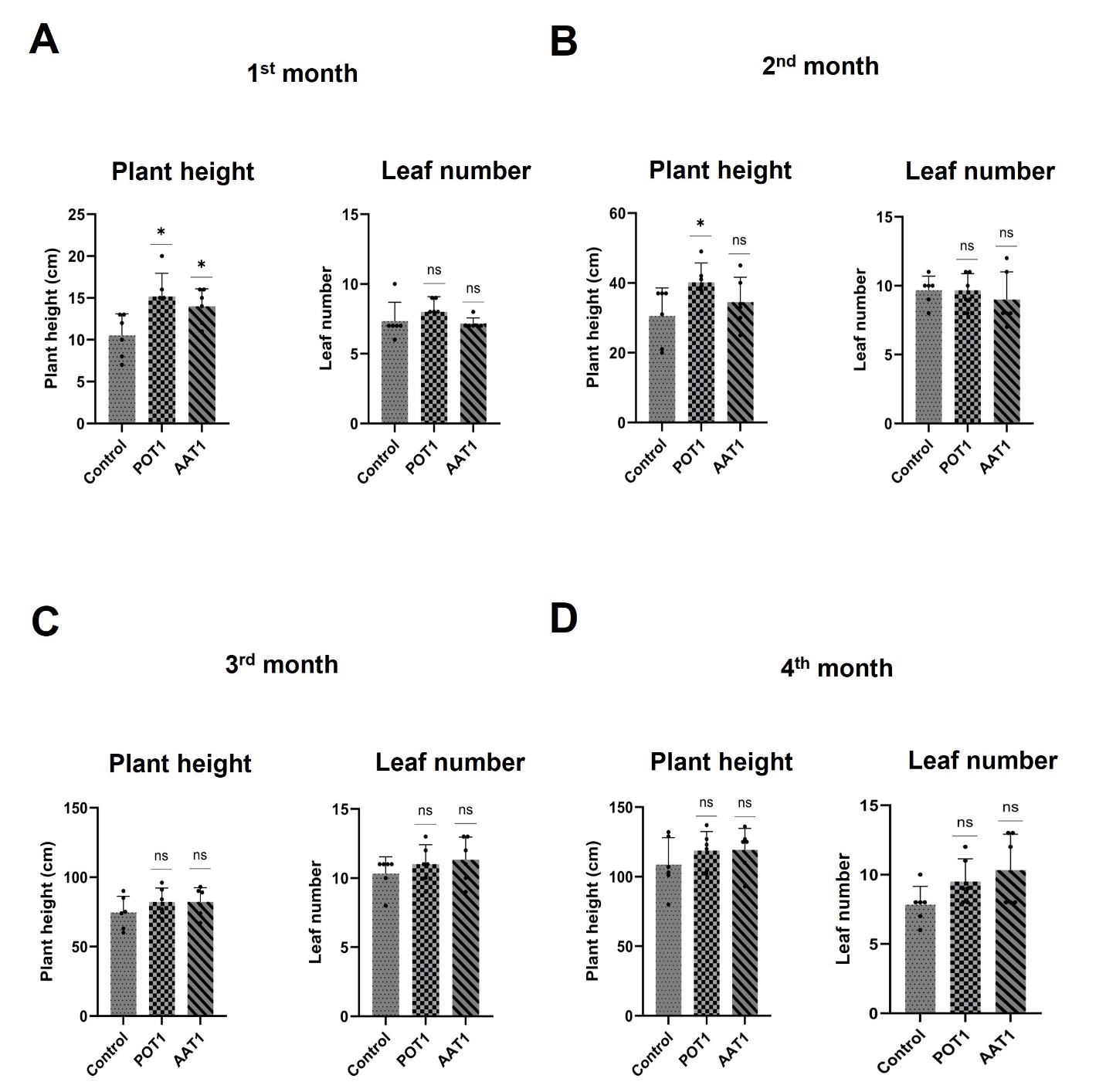


**FIG S6.** Effect of colonization on growth characteristics of sengon by *P. olsonii* TLL1 (POT1) and *A. aculeatus* TLL1 (AAT1) during initial growth. Quantitative analysis of various growth parameters: plant height and leaf number at (A) 1^st^ month, (B) 2^nd^ month, (C) 3^rd^ month, and (D) 4^th^ month post inoculation. Data are presented as mean ± SD, with six biological replicates. Statistical significance was determined using unpaired two-tailed *t*-test, with significance levels indicated by * *p* < 0.05, ** *p* < 0.01, *** *p* < 0.001.


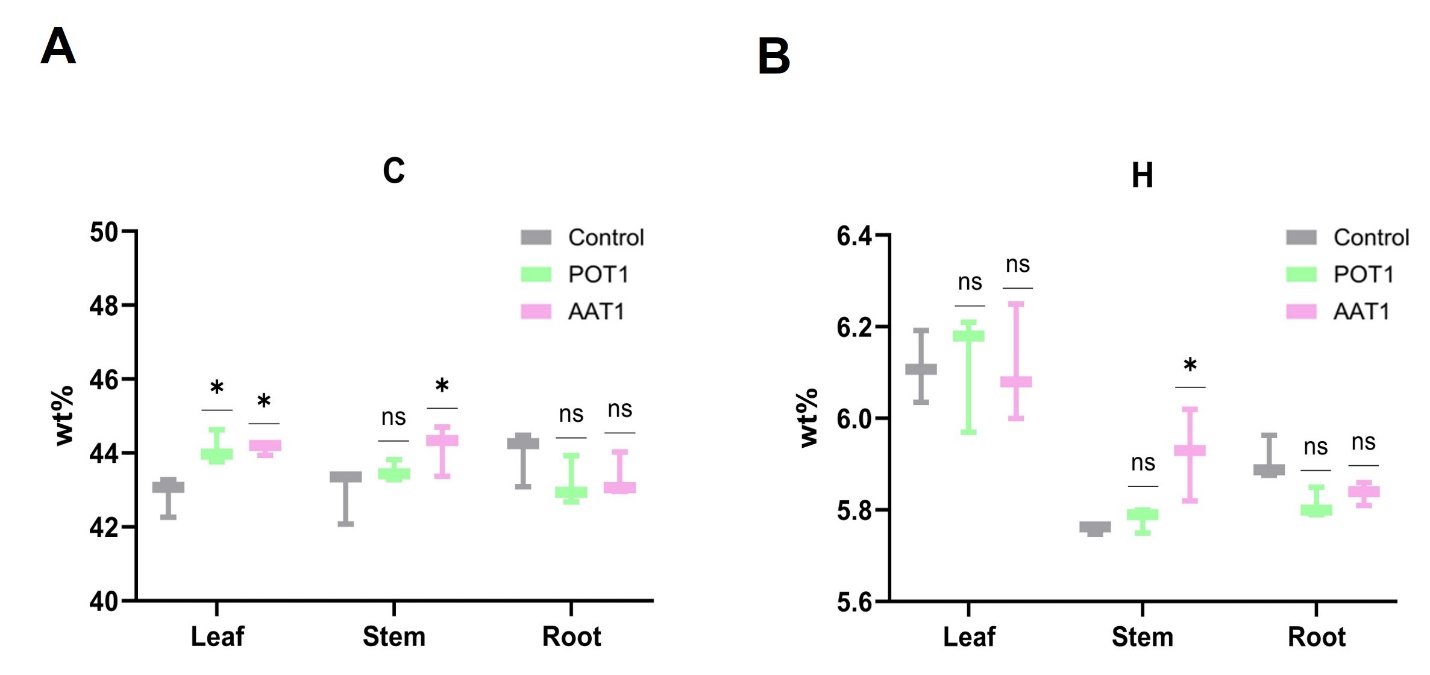


**FIG S7.** Quantitative analysis of nutrient elements in sengon treated with *P. olsonii* TLL1 (POT1) and *A. aculeatus* TLL1 (AAT1). Elemental analysis of the nutrients: carbon (C), and hydrogen (H) in leaf, stem, and root of the treated and uninoculated plants are shown. Data are presented as mean ± SD, with three biological replicates. Statistical significance was determined using two-way ANOVA.


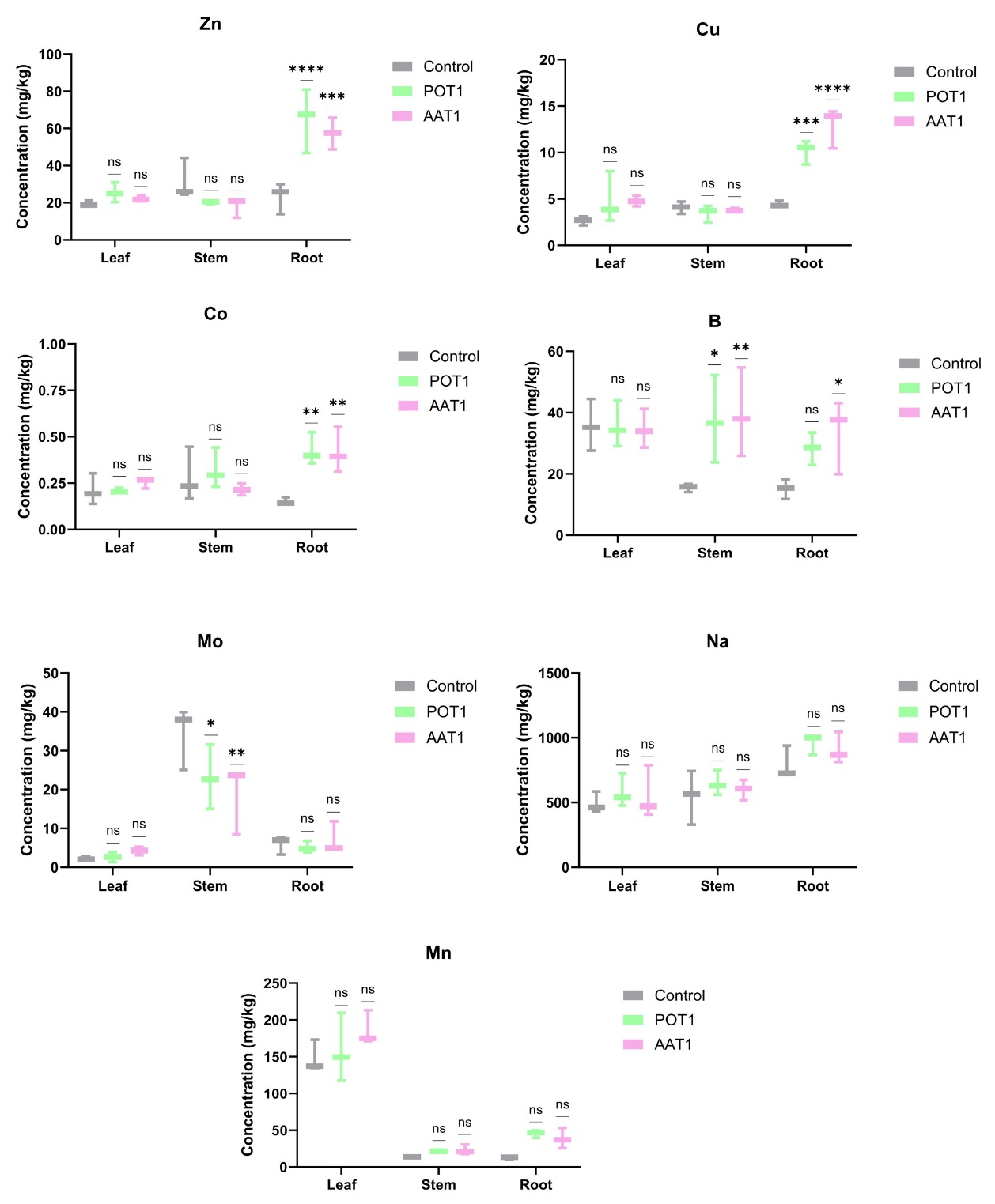


**FIG S8.** Analysis of micronutrient elements in sengon treated with *P. olsonii* TLL1 (POT1) and *A. aculeatus* TLL1 (AAT1). Quantitative analysis of the nutrients: boron (B), copper (Cu), molybdenum (Mo), manganese (Mn), sodium (Na), zinc (Zn), and cobalt (Co) in leaf, stem, and root of the treated and uninoculated plants is shown. Data are presented as mean ± SD, with three biological replicates. Statistical significance was determined using two-way ANOVA.


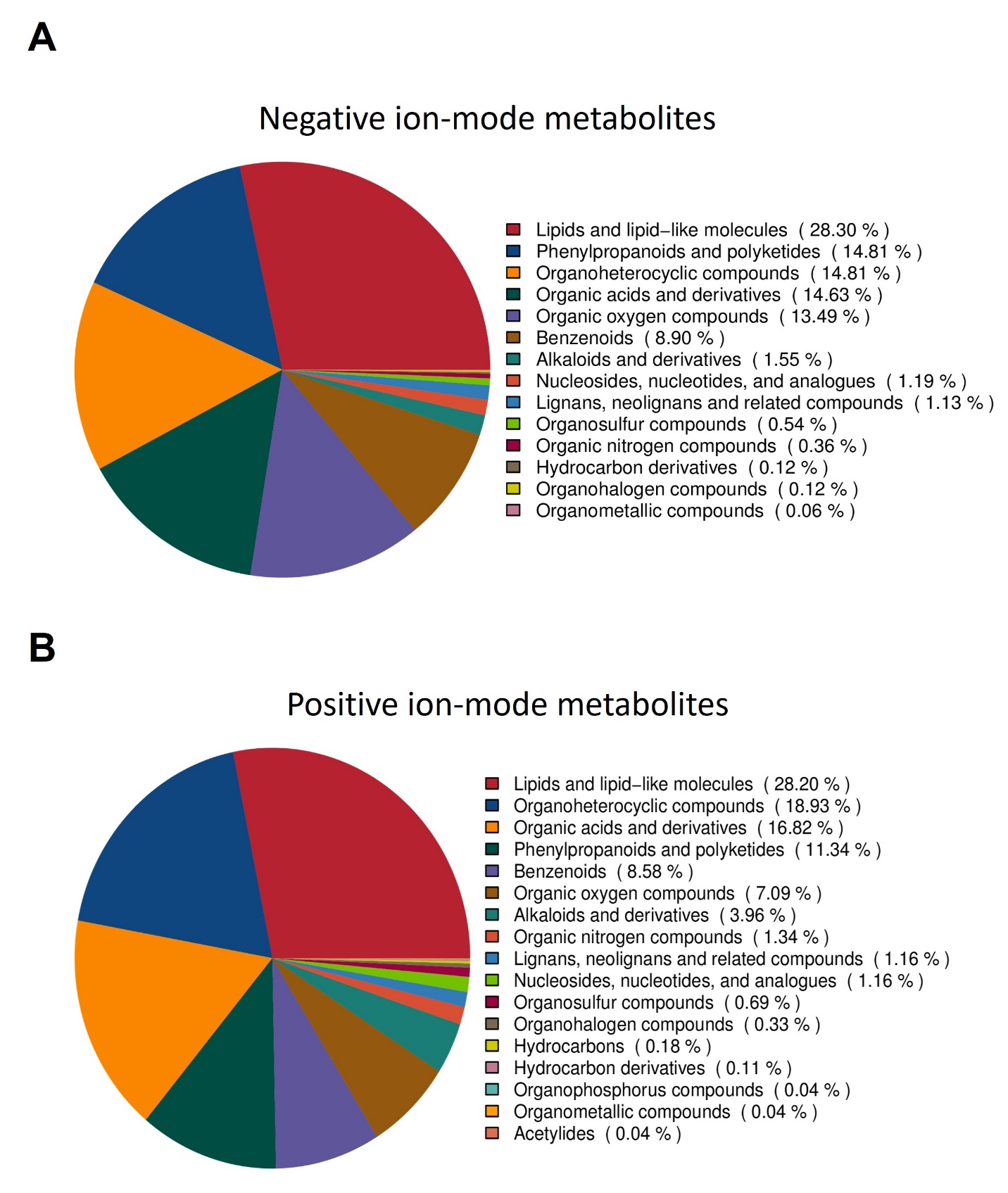


**FIG S9.** Chemical classification of metabolites in sengon colonized by *P. olsonii* TLL1 (POT1) and *A. aculeatus* TLL1 (AAT1). Classification of metabolites identified in (A) negative and (B) positive ion mode metabolomic analysis are shown.
